# YKL-40 levels are independently associated with albuminuria in type 2 diabetes

**DOI:** 10.1186/1475-2840-10-54

**Published:** 2011-06-22

**Authors:** Anne K Røndbjerg, Emina Omerovic, Henrik Vestergaard

**Affiliations:** 1 Center of Endocrinology and Metabolism, Dept. of Medicine O, Copenhagen University Hospital Herlev, Denmark; 2 Faculty of Health Sciences, University of Copenhagen, Denmark

**Keywords:** Type 2 diabetes, biomarker, nephropathy

## Abstract

**Objective and design:**

YKL-40 is involved in inflammation and endothelial dysfunction, and is increased in patients with type 1 diabetes, with an independent association between increasing YKL-40 levels and increasing levels of albuminuria. YKL-40 is associated with atherosclerosis and an increased cardiovascular mortality in the general population. In the present study YKL-40 levels were examined in patients with type 2 diabetes (T2D) with increasing levels of albuminuria, known to be associated with an increased risk of cardiovascular disease.

**Materials and methods:**

One-hundred-five patients with T2D were examined: 49 with normoalbuminuria (N, U-albumin/creatinine < 2.5 mg/mmol), 35 with persistent microalbuminuria (MA, 2.5-25 mg/mmol) and 21 with persistent macroalbuminuria/diabetic nephropathy (DN, > 25 mg/mmol). The control group consisted of 20 healthy individuals (C). Groups were matched according to age, gender and known duration of diabetes.

**Results:**

Median levels (interquartile range) of serum YKL-40 were significantly higher in N and MA vs. C (86 (55-137) ng/ml and 84 (71-147) ng/ml, respectively vs. 41 (33-55) ng/ml, p < 0.01) and even higher in patients with DN (120 (83-220) ng/ml, p < 0.001 for all comparisons). YKL-40 levels correlated with urinary albumin/creatinine-ratio in the total group of participants (r = 0.41, p < 0.001). Significant intercorrelations of YKL-40 were found with age, duration of diabetes, systolic blood pressure, lipid levels, HbA1c and HOMA-IR. After adjustment for significant covariates, albuminuria was significantly associated with YKL-40 levels (r = 0.32, p = 0.006).

**Conclusions:**

YKL-40 levels are elevated in patients with T2D with an independent association between increasing YKL-40 levels and increasing levels of albuminuria. The study suggests a role of YKL-40 in the progressing vascular complications in patients with T2D.

## Introduction

Albuminuria is a well-established independent predictor for diabetic nephropathy and is also a strong predictor for cardiovascular morbidity and mortality in both patients with type 1 (T1D) and type 2 diabetes (T2D) [1-4]. The exact mechanisms between albuminuria and cardiovascular disease are still unclear, but it is suggested that increasing levels of albuminuria reflect vascular damage in the kidneys as part of a systemic endothelial dysfunction, which is the initial step in atherogenesis [5]. Individuals with diabetes have in general a 2- to 4-fold increased risk of subsequent cardiovascular disease (CVD)[6].

YKL-40 is a glycoprotein involved in inflammation and endothelial dysfunction. It is a growth factor for various cell types and has an important role in extracellular matrix remodeling and angiogenesis [7,8]. In the last few years, several clinical studies have described elevated YKL-40 levels in several cardiovascular conditions, as well as described an association between YKL-40 and mortality. YKL-40 has been found to be associated with all-cause as well as cardiovascular mortality in both patients with stable ischemic heart disease (IHD) [9] and in the general population above 50 years of age without known diabetes or IHD [10]. YKL-40 levels are elevated both in patients with T1D and T2D known to be at high risk for the development of cardiovascular diseases [10-12], and in patients with T1D increasing levels of YKL-40 are seen with increasing levels of albuminuria, suggesting that YKL-40 might be able to be used as an early marker of CVD [11].

The objective of the present study was to evaluate serum YKL-40 in patients with T2D and with increasing levels of albuminuria.

## Materials and methods

### Study population

One-hundred-five patients with T2D were examined: 49 with normoalbuminuria (N, U-albumin/creatinine < 2.5 mg/mmol), 35 with persistent microalbuminuria (MA, 2.5-25 mg/mmol) and 21 with persistent macroalbuminuria/diabetic nephropathy (DN, > 25 mg/mmol). The control group consisted of 20 healthy individuals (C). Groups were matched according to age, gender and known duration of diabetes.

### Measurements

Plasma samples were taken as part of the first routine visit (inclusion) in the morning after an overnight fast and were stored at - 80°C until analysis. Beside routine analyses, analyses of serum YKL-40 were measured using ELISA method (Quidel, USA). Measuring range was 20-300 ng/ml, with intra- and interassay coefficients of variation of 5.8% and 6.0%. Glomerular filtration rate (GFR) was estimated using the 4 variable Modification of Diet in Renal Disease GFR formula (age, gender, race, serum creatinine) http://mdrd.com/. Insulin resistance was estimated using the HOMA model http://www.dtu.ox.ac.uk/homacalculator/index.php. Urinary albumin was measured by immunoturbidimetry on a Cobas Bioanalyzer (Roche Products, Switzerland) from early morning spot urine collections. Lower detection limit was 1 mg/l, coefficients variations < 4.0%. Urinary creatinine was measured by a Jaffé reaction rate, using a kinetic principle to eliminate pseudocreatinines. Urinary albumin excretion was determined as UACR. The degree of albuminuria was confirmed by at least 2 consecutive tests. Diabetic retinopathy was assessed in most patients by fundus photography after pupillary dilatation and graded as nil, simplex or proliferative retinopathy.

The study was approved by the Danish Data Protection Agency (id. 30122009.HEH.O.JF) and the local ethics committee of Copenhagen (KA 03065) and investigations conformed to the principles of The Helsinki Declaration.

### Statistical analyses

Following a test of statistical normality, data values are presented as mean ± SD or as median and interquartile range (IQR). For continuous variables, comparisons between the group of patients with T2D and the group of control subjects were performed with One-Way ANOVA. Mann-Whitney test was used if Levene's test for equality of variance was significant, or if a variable exhibited a clear non-Gaussian distribution. If data had a non-Gaussian distribution data were logarithmically transformed. Analyses of intercorrelations and associations were performed using univariate and multivariate linear regression analysis. The χ2-test was used for categorical variables.

P values were two-sided, and p < 0.05 was considered statistically significant. All analyses were made with the statistical software package SPSS (version 11.5 SPSS; Chicago, IL).

## Results

**Figure 1:**
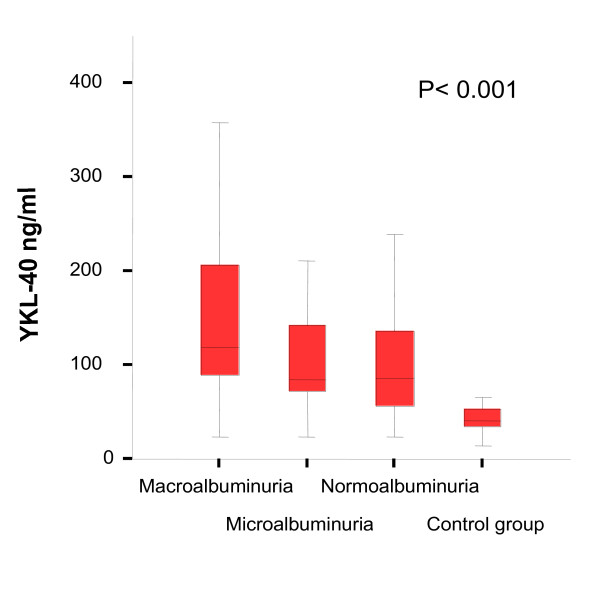
**Median levels (interquartile range) of serum YKL-40 were significantly higher in N and MA vs. C (86 (55-137) ng/ml and 84 (71-147) ng/ml, respectively vs. 41 (33-55) ng/ml, p < 0.01) and even higher in patients with DN (120 (83-220) ng/ml, p < 0.001 for all comparisons)**.

Clinical data for the control group and the T2D patients with different levels of albuminuria is shown in Table 1. The groups were closely matched according to age, gender and duration of diabetes. YKL-40 levels according to level of albuminuria are illustrated in Figure 1.

**Table 1:** Clinical data of control group and the type 2 diabetic patients differentiated according to level of albuminuria

	Control group	Normoalbuminuria	Microalbuminuria	Macroalbuminuria	p value
N, total	20	49	35	21	
Male (%)	60.4	44.8	34.3	42.3	0.24
Age (years)	57.1 (7.2)	61.3 (12.0)	60.1 (11.7)	64 (13.1)	0.23
DM duration (years)	-	5.0 (0.3-10.0)	6.0 (0.2-10.0)	5.5 (3.3-9.8)	0.65
BMI (kg/m^2^)	28.9 (2.9)	29.9 (5.7)	33.9 (7.4)	30.9 (5.7)	0.02
Smoking	2 (10.0%)	19 (38.7%)	19 (49.0%)	8 (38.1%)	0.25
Use of antihypertensiva	0	31 (63.2%)	28 (80.0%)	19(90.5%)	< 0.001
Use of statins	1 (5.0%)	33 (67.3%)	21 (60.0%)	13 (61.9%)	< 0.001
Use of aspirin	1 (5.0%)	24 (49.0%)	10 (30.0%)	12 (57%)	< 0.001
Use of metformin	-	30 (61.2%)	16 (45.7%)	14 (66.7%)	< 0.001
Use of SU	-	20 (40.8%)	12 (34.2%)	8 (38.1%)	0.008
Use of Insulin	-	7 (14.2%)	6 (17.1%)	5 (23.8%)	0.14
					
YKL-40 (ng/ml) *	41 (33-55)	86 (55-137)	84 (71-147)	120 (83-220)	< 0.001
HbA1c (%) **	5.6 (0.3)	8.6 (2.1)	8.0 (2.0)	7.9 (1.8)	< 0.001
Creatinine (μmol/l) **	85 (76-94)	75 (65-93)	80 (65-91)	106 (66-121)	0.45
UACR (mg/mmol) *†	0.57 (0.34-1.53)	0.68 (0.39-1.23)	6.65 (4.06-11.47)	49.3 (36.9-102.9)	< 0.001
eGFR (ml/min/1.73 m^2^) **	78.2 (25.7)	81.6 (21.9)	83.8 (24.3)	73.4 (32.3)	0.61
Cholesterol (mmol/l)	5.6 (0.9)	4.5 (1.2)	4.6 (1.0)	4.6 (1.1)	0.001
Systolic BP (mmHg) **	142 (17)	137 (18)	140 (16)	145 (17)	0.23
Diastolic BP (mmHg) **	85 (12)	81 (8)	83 (11)	83 (11)	0.34
History of:					
CVD (all)	0	11 (22.4%)	5 (14.3%)	7 (33.3%)	0.03
Myocardial infarction	0	5 (10.2%)	3 (8.6%)	1 (4.7%)	0.49
Stroke	0	5 (10.2%)	2 (5.7%)	4 (19.0%)	0.11
PAOD	0	3 (6.1%)	1 (2.8%)	3 (14.3%)	0.13
					
Retinopathy:					
- none	-	36 (73.5%)	30 (85.7%)	10 (47.6%)	0.002
- simplex	-	2 (4.1%)	2 (5.7%)	3 (14.2%)	0.002
- proliferative	-	0	0	3 (14.2%)	0.002

Presented as numbers (%) where not specified. * Median (IQR). ** Mean (SD). † Most of the patients had UACR levels reduced by antihypertensive medication which was not stopped when spot urine samples were collected for the study.

HbA1c, hemoglobin A1c; UACR, urinary albumin/creatinine ratio; eGFR, estimated glomerular filtration rate (MDRD); BP, blood pressure; PAOD, peripheral arterial occlusive disease

Median YKL-40 levels were significantly different between groups with increasing YKL-40 levels with increasing levels of albuminuria (p < 0.001). We found no difference in eGFR between the different groups (p = 0.61). We did find a significant difference in UACR between the different groups (p < 0.001), even though most of the patients had UACR levels already reduced by antihypertensive medication, which was not stopped when spot urine samples were collected for the study. There was a significant difference in use of antihypertensive drugs between the groups (p < 0.001), with as expected more patients treated in the micro- and macroalbuminuric groups.

We found no significant difference in systolic or diastolic blood pressure between the groups for all comparisons or comparisons of the diabetic groups. We did find significant differences in cholesterol and HbA1c levels (p < 0.001) between all groups due to differences between controls and the diabetic groups in total. No significant differences in cholesterol or HbA1c were found the diabetic groups in between.

**Figure 2:**
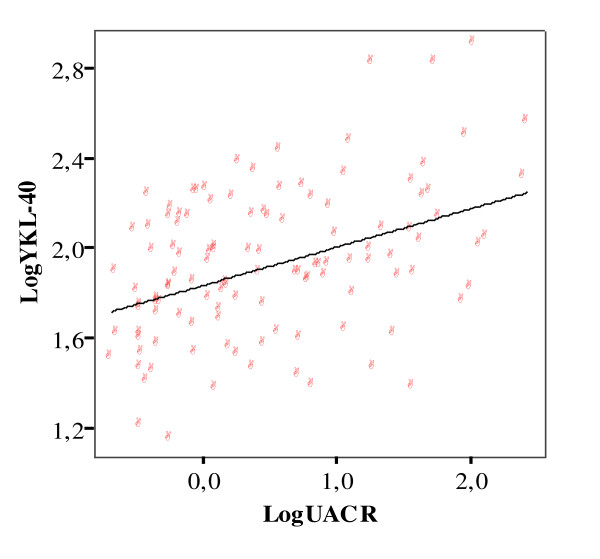
**Association between log-transformed UACR and YKL-40 levels (r = 0.41, p < 0.0001)**.

Simple and multiple regression analyses showed correlation of YKL-40 with UACR in the total group of participants (r = 0.41, p < 0.001) (Table 2, Figure 2). Significant intercorrelations of YKL-40 were also found with age, HOMA-IR, HbA1c, total cholesterol, LDL-cholesterol, triglycerides, duration of diabetes, and systolic blood pressure (0.001 < p < 0.047) (Table 2).

**Table 2:** Intercorrelations of YKL-40

	Correlation coefficient	P
UACR	0.41	< 0.001
Age	0.32	< 0.001
HOMA-IR	0.31	0.004
HbA1c	0.28	0.004
Triglycerides	0.23	0.011
Systolic blood pressure	0.20	0.029
Duration of diabetes	0.20	0.045
Total cholesterol	-0.25	0.006
LDL-cholesterol	-0.20	0.047

Log-transformed data

In a multiple regression model adjusting for the significant covariates (UACR, age, HOMA-IR, HbA1c, triglycerides, systolic blood pressure, duration of diabetes, total cholesterol and LDL-cholesterol), albuminuria was still found to be significantly associated with YKL-40 levels (r = 0,32, p = 0.006). Moreover, significant associations were also found with total cholesterol (r = 0.52, p = 0.024) and LDL-cholesterol (r = -0.53, p = 0.021).

UACR was significantly associated with YKL-40, BMI, HbA1c and systolic blood pressure (0.001 < p < 0.02). A multiple regression analyses adjusted for significant covariates demonstrated that YKL-40 was associated with albuminuria (r = 0.42, p < 0.001).

More patients with T2D were treated with statins, when compared to the control subjects (p < 0.001), but there were no differences in the number of T2D treated between the groups. No differences were seen in levels of YKL-40 in the whole group of T2D patient treated with statins when compared to non-treated patients (p = 0.18), nor in the individual groups (0.1 < p < 0.9).

At baseline, only a limited number of subjects had a history of myocardial infarction, stroke or symptoms of intermittent claudicatio. No differences in YKL-40 levels were found in patients with a history of CVD (any), when compared to patients without these macrovascular complications (p = 0.47). However, more patients with macroalbuminuria had CVD (χ^2 ^= 8.6, p = 0.036). Patients with retinopathy had significantly higher levels of YKL-40 (p = 0.01), only due to differences between normoalbuminuric and microalbuminuric patients (p = 0.021). More patients with albuminuria had retinopathy (χ^2 ^= 23.6, p = 0.001).

## Discussion

Micro- and macrovascular complications are known to decrease the quality of life as well as shorten the life expectancy of patients suffering from diabetes. Despite intensive research in the pathological mechanisms resulting in improved and intensified treatment of diabetes and its vascular risk factors and complications, there is still a need for supplementary risk markers to understand the pathogenesis and predict the development of micro- and macrovascular disease.

We evaluated levels of YKL-40, a marker of inflammation and endothelial dysfunction, in patients with T2D. We found significant elevated YKL-40 levels in patients with T2D compared to healthy control subjects, and significant increased YKL-40 levels with increasing levels of albuminuria. This finding is in accordance with previous studies showing that chronic low-grade inflammation is associated with the occurrence and progression of microalbuminuria [13], and that both micro- and macroalbuminuria are accompanied by increased levels of a variety of markers of endothelial dysfunction [14]. Our findings are also in accordance with previous studies showing that YKL-40 levels are elevated in both T2D and T1D patients, when compared to control subjects, and for T1D patients with microvascular disease [10-12,15]. Rathcke et al found that YKL-40 levels were elevated in patients with T1D and increased with levels of albuminuria, and that YKL-40 levels were independently associated with increasing levels of albuminuria after adjustment for significant covariates [12], comparable to the results of this study of T2D patients and control subjects.

Similar to our findings, 2 recent studies have shown significant elevation of YKL-40 in T2D patients with albuminuria [16,17]. In a brief report from Japan, increased YKL-40 levels were found in normal weight or slightly overweight patients with T2D with different levels of albuminuria [17]. Regression analyses demonstrated, as in our study, an association between YKL-40 and albuminuria, and that serum YKL-40 was a determinant of albuminuria independently of conventional risk factors [17]. No control group was included in the Japanese study and the duration of diabetes was approximately 16 years, in the present study 5.5 years, which may influence YKL-40 levels as seen in our study, and nothing was mentioned about medication and CVD. In a study from Austria, comparable results were found, but again no control group was included and the duration of diabetes was 11-14 years, and the percentage of patients with any CVD was between 75-82%, in our study 11-33%, implying more advanced CVD in the T2D patients from Austria [16].

The association found between YKL-40 and albuminuria in both T1D and T2D could reflect common determinants, such as inflammation, or a causal link where inflammation leads to increase in YKL-40, and subsequent generalized vascular damage reflected by albuminuria. YKL-40 is excreted by the kidney and this may affect the predictive ability of the biomarker in patients with impaired kidney function. Not surprisingly we found a significant correlation between YKL-40 and UACR, but we did not find that increasing YKL-40 levels were predicted by a decline in eGFR. Despite the correlation between YKL-40 and UACR, we found a significant association between YKL-40 levels and level of albuminuria after adjustment for significant covariates, implicating increasing albuminuria with increasing YKL-40 levels.

In a previous study we found an association between YKL-40 and an increased cardiovascular (CV) mortality rate in an elderly part of the general population without diabetes and CVD, after adjustment for known CVD risk factors and markers [10]. YKL-40 and UACR were independent markers of CVD mortality with only weak intercorrelation and in accordance with the studies on low-grade albuminuria and risk of CVD, YKL-40 and low-grade albuminuria had a synergistic prediction of CVD mortality. Other studies have supported the association between YKL-40 and CVD morbidity and mortality, showing that YKL-40 levels are associated with the presence and extent of coronary artery disease as assessed by coronary angiography [18]. Moreover YKL-40 levels have been found to be elevated in patients with myocardial infarction [19].

It has been demonstrated that statins, through their capacity to inhibit inflammation, can reduce the increased inflammatory activities seen with progression of atherosclerosis, and this is correlated with the reduction in calcification [20]. In the present study we found that significantly more T2D patients were treated with statins, and no differences were found between the diabetic groups. Because of the higher levels of YKL-40 seen in T2D patients, we cannot exclude that statin treatment has influenced the levels of YKL-40, as seen in a previous study demonstrating reduced YKL-40 in statin compared to non-statin treated atherosclerotic patients [21]. However, since statin treatment reduces inflammation and can ameliorate the calcification response in the vessel wall, higher YKL-40 levels would be expected had the patients been without statin treatment. However, no differences were found in levels of YKL-40 between statin treated and non-treated patients.

Vascular endothelial dysfunction is an important factor in the pathogenesis of diabetic micro- and maroangiopathy [14]. YKL-40 promotes chemotaxis, cell attachment, spreading, and migration of vascular endothelial cells, suggesting that YKL-40 promotes the process of atherosclerotic plaque formation, in which vascular smooth muscle cells (VSMCs) are induced to migrate through the intima in response to exogenous signals [7]. YKL-40 also participates in the modulation of vascular endothelial cell morphology by promoting the formation of branching tubules, indicating a role of YKL-40 in angiogenesis by stimulating the migration and reorganization of VSMCs [7]. Moreover, YKL-40 is produced and secreted by monocytes during differentiation to macrophages but is also secreted by activated macrophages, and YKL-40 protein expression is found in vivo in both macrophages and VSMCs in the atherosclerotic plaque [8]. In accordance with this finding, both T1D and T2D patients have been found to have increased monocytic activity characterized by increased monocytic release of proinflammatory cytokines and reactive oxygen species, which is accentuated in T1D patients with microvascular complications [22]. Therefore, it seems that YKL-40 participates in monocyte differentiation and macrophage activation as part of the endothelial dysfunction and the processes during early stages of atherosclerosis [8]. YKL-40 seems to be of pathogenic importance in the low-grade inflammation that precedes the development of CVD.

YKL-40 also seems to promote tumor angiogenesis in colon and breast cancer [23], an effect that could point to a common pathway for several disease processes, where YKL-40 is expressed in monocytes, stimulates monocyte maturation and differentiation to macrophages, and stimulates macrophage activation and promotes angiogenesis. This cascade can be identified in the pathology of atherosclerosis, diabetic complications and cancers. However, it still remains to be elucidated whether YKL-40 is a biomarker of general inflammation or is more directly involved in specific disease processes.

There are limitations of the present study. We have performed a cross-sectional analysis of YKL-40 levels in relation to albuminuria in patients with T2D; however long-time studies are needed to clarify whether an increase in YKL-40 levels in both T1D and T2D patients is a consequence of a general progressing atherosclerotic disease, or whether YKL-40 can predict or contribute to microvascular and macrovascular disease in these patients. Moreover, in the present study no other markers of endothelial dysfunction and inflammation were investigated, e.g. high-sensitive C-reactive protein (hsCRP). But in contrast to most inflammatory markers, YKL-40 is locally produced at the site of inflammation [12,24] and previous studies have either not found or have found only a weak correlation with C-reactive protein [10,12]. Perception of YKL-40 as an early marker [10,24] indicates, that YKL-40 could possibly correlate to other early markers of endothelial activation and/or dysfunction.

In conclusion, YKL-40 levels are elevated in patients with T2D with an independent association between increasing YKL-40 levels and increasing levels of albuminuria after adjustment for UACR, age and other significant covariates. A role of YKL-40 in the gradually progressing vascular complications in patients with diabetes is suggested, with YKL-40 being a possible early marker of the widespread atherosclerotic complications in diabetic patients.

## Competing interests

The authors declare that they have no competing interests.

## Authors' contributions

AKR and EO participated in the design, coordination of the study, and in analysis and interpretation of data and drafted the manuscript. HV conceived of the study and participated in the design, coordination, conduct of the study, performed the statistical analysis and helped to draft the manuscript. All authors have read and approved the final version of the manuscript

## Disclosure

None of the authors declare any financial disclosures.
